# Effectiveness, safety and indications of acute normovolemic haemodilution in total knee arthroplasty

**DOI:** 10.1038/s41598-024-53779-6

**Published:** 2024-02-08

**Authors:** Yucong Li, Jingle Chen, Hao Xie, Hangxing Wu, Zhijie Zuo, Wanyan Hu, Chao Xie, Lijun Lin

**Affiliations:** Department of Joint and Orthopedics, Zhujiang Hospital, Southern Medical University, Guangzhou, Guangdong People’s Republic of China

**Keywords:** Acute normovolemic haemodilution, Total knee arthroplasty, Perioperative blood management, Indications, Orthopaedics, Clinical trial design

## Abstract

Total knee arthroplasty (TKA) is the most cost-effective, and potent method for the treatment of end-stage knee osteoarthritis. Acute normovolemic haemodilution (ANH) can effectively replace the need for allogeneic transfusions due to the high amount of bleeding during TKA. However, more studies are needed to prove the efficacy and safety of ANH and to clarify its indications in the field of knee replacement. Medical records from June 1, 2019 to June 1, 2021 were searched and grouped according to inclusion and exclusion criteria. PART I: 58 patients with ANH during TKA were selected as the ANH group (n = 58), and 58 patients with allogeneic transfusion were chosen as the control group (n = 58). PART II: Patients with anaemia were divided into the ANH group (n = 18) and the control group (n = 12). PART I: The postoperative inflammatory index and serum albumin in the ANH group were significantly lower than those in the control group. No significant difference was observed in the theoretical loss of red blood cells, postoperative renal function, liver function, cardiac function and biochemical ion index between the two groups. The effective rate of ANH in the normal haemoglobin group was significantly lower than that in the anaemia group. PART II: In patients with anaemia, the theoretical loss of red blood cells in patients with ANH was less than that in the control group. The postoperative inflammation, renal function, liver function and cardiac function in the ANH group were better than those in the control group, and no significant difference was noted in biochemical ions and nutritional status indicators. This paper shows that ANH not only can replace allogeneic transfusion in TKA, especially in patients with anaemia, but also has lower inflammatory indicators than allogeneic transfusion. From a security perspective, the body’s tolerance to ANH is within the body’s compensation range.

## Introduction

Osteoarthritis (OA) is a common degenerative joint disease with clinical manifestations including pain, limited mobility and deformity^1^. Total knee arthroplasty (TKA) is currently the most cost-effective method for the treatment of advanced knee osteoarthritis^2^. Moreover, TKA is one of the most common major orthopaedic surgeries according to the level of surgery. As a major orthopaedic surgery, perioperative haemorrhage in TKA places patients at risk of blood transfusion^3^. Blood loss of 1000–1500 mL has been reported in patients receiving TKA, with 10–38% requiring subsequent allogeneic transfusion^3–6^. Although allogeneic transfusion is the most common method, it has potential risks similar to allogeneic organ transplantation, may have long-term effects on immunity and may lead to micro thrombosis, coagulation or hemolysis^7^. Thus, allogeneic transfusion should be used as an independent tool for the development of postoperative infection predictors^7,8^.

Anaemia is strongly associated with TKA. According to the WHO criteria for anaemia diagnosis and efficacy, a survey of 57,664 residents showed the incidence of anaemia amongst people aged 60 and over in China was 31.4%, and the incidence of anaemia increased with age. Spahn included 19 prospective or retrospective studies; the results showed the incidence of postoperative anaemia in 29,068 patients with TKA was 83.9%, and the average postoperative Hb decreased by 24 g/L^9^. According to the evaluation in our hospital, the theoretical haemorrhage of patients with TKA in our department is 2.41 U after an operation. In addition, the haematocrit of the elderly is low, and the theoretical bleeding can be 700 mL, accounting for about 20% of the blood. Moreover, perioperative anaemia brings inconvenience to patients with TKA: Firstly, perioperative anaemia state can prolong the hospital stay of patients^9–11^. Secondly, preoperative anaemia and postoperative anaemia significantly increase postoperative mortality^9,12^. Thirdly, higher Hb levels after orthopaedic surgery promote patients’ functional recovery, and anaemia is an important factor of postoperative activity and functional recovery, which is an independent risk factors for functional activity and normal walking^10,13^. Furthermore, postoperative Hb level is positively correlated with patients’ quality of life score at two months after surgery^14^.

Since 1980, allogeneic blood transfusion (ABT) has been widely recognised by clinicians for its advantages of preventing disease transmission, less adverse reactions, saving blood resources, safety and efficiency, and has been widely used in the treatment of anaemia and surgical bleeding^15,16^. Acute normovolemic haemodilution (ANH) is a potentially useful blood conservation strategy^17^. Because autologous blood is ultimately transfused, this simple technique does not require compatibility testing and is not associated with infectious disease transmission. In addition, the decreased blood viscosity and the consequent increase in blood flow associated with NVHD is likely protective against postoperative thromboembolic complications^18^. In recent years, ANH, as a method of ABT, has been widely used in major operations such as the cardiovascular system and digestive systems, and its efficacy and safety have been proven^19–21^. However, data regarding the compensatory capacity of elderly patients during ANH are limited^22^. Then, whether ANH can also be used in TKA has become a problem that needs to be solved.

## Methods

### Setting and subjects

This study was completed in the Department of Joint and Osteopathy, Zhujiang Hospital, Southern Medical University from June 1, 2019 to June 1, 2021. The electronic medical records of hospitalised patients who underwent TKA were reviewed. The exclusion criteria were as follows: undergoing nonunilateral TKA surgery, aged < 60 years or > 80 years; with severe coagulation disorders, low platelet count (Plt) or abnormal platelet function; with severe heart, lung, liver and kidney failure; with systemic diseases such as rheumatism; with severe infection before surgery. Our department started to implement ANH on June 1, 2019 for patients with knee osteoarthritis who are planning to undergo TKA surgery. Patients with TKA who received ANH between June 1, 2019 and June 1, 2021 were included in the experimental group (n = 58), and the same number of patients with TKA who received allogeneic transfusion (400 mL) were randomly selected and included in the control group (n = 58).

### Treatment

Shortly after anaesthesia administration, ANH was performed by a skilled attending anaesthesiologist under ECG, oxygen saturation and invasive radial artery blood pressure monitoring. A predetermined amount of autologous blood was extracted, and an equal amount of colloid (6% hetastarch, middle molecule, 140/0.4) was injected at the same time. ANH blood was collected into a 400 mL citrate dextrose phosphate collection bag and stored at room temperature. Half an hour before surgery, cefuroxime was dripped intravenously at 1.5 g/100 mL. Then, 2 g/100 mL tranexamic acid was administered intravenously. All operations were performed by Professor Lijun Lin. Stryker Triathlon knee systems was used for surgical prostheses. All operations were performed according to the standard of knee arthroplasty. The skin and joint capsule were cut through the medial parapatellar approach commonly used in the clinic to expose the knee joint. Subsequently, femoral osteotomy and tibial plateau osteotomy were performed, PS implants were installed, and joint capsule and skin were sutured. Autologous blood was returned before the air pressure tourniquet was released after the surgical prosthesis was placed or when Hb reached the threshold of ≤ 70 g/L.

### Demographic and baseline parameters

Baseline demographic variables, including weight, sex and height, were collected. Surgical modality and baseline laboratory data were also recorded: Hb, haematocrit (Hct), Plt, white blood cell (WBC), erythrocyte sedimentation rate (ESR-T), high sensitivity C-reactive protein (HSCRP), albumin (Alb), glucose (Glu), creatinine (Cr), urea nitrogen (Urea), estimated glomerular filtration rate (eGFR), cystatin C (Cys-C), glutamyl transferase (GGT), aspartate transaminase (AST), alanine transaminase (ALT), rate-pressure-product (RPP), diastolic pressure (DP), VAS pain grade (VAS), range of motion of knee joint (ROM) and The Western Ontario and McMaster Universities osteoarthritis index (WOMAC).

### Outcomes

The primary outcome of this retrospective study was the difference in theoretical red blood cell loss (TRBCL) between the two groups [TRBCL = preoperative blood volume (PBV) × [preoperative haematocrit (preHct) − postoperative haematocrit (postHct)]. AND PBV = K1 × [height(m)]^3^ + K2 × weight (kg) + K3; K1 = 0.3669, K2 = 0.03219 and K3 = 0.6041 for males; and K1 = 0.3561, K2 = 0.03308 and K3 = 0.1833 for females]^23^. Secondary outcomes were perioperative blood routine index (Hb, Hct and Plt), inflammatory index (WBC, HSCRP and ESR-T), biochemical index (Glu and Alb), renal function index (Cr, Urea, e-GFR and Cys-C), liver function index (GGT, AST and ALT), cardiac function index (RPP and DP) and changes and rehabilitation index (VAS, ROM and WOMAC). Postoperative adverse event data, including pulmonary complications, serious wound, abscess, infection, dehiscence, bleeding requiring reoperation, ischemic events (myocardial/cerebral infarction) and acute kidney injury (AKI) were also collected. The relationship between these perioperative variables and theoretical haemorrhage was examined by univariate analysis and multiple linear regression analysis.

### Sample size

Part 1: We performed simple random sampling and the results were as follows: ANH group (n = 10, 1.88 ± 0.39U) and Control group (n = 10, 2.12 ± 0.35U). The α (test level) = 0.05 (two-sided test), and 1-β (test power) = 0.8. Based on the assumption of homogeneity of variance, Pooled varance (Sp2) was used to calculate the sample size. The data were imported into PASS 11.0 software and the result was N1 = N2 = 39.

Part 2: We performed simple random sampling and the results were as follows: ANH group (n = 5, 1.26 ± 0.54U) and Control group (n = 5, 1.98 ± 0.59U). The α (test level) = 0.05 (two-sided test), and 1 − β (test power) = 0.8. Based on the assumption of homogeneity of variance, Pooled varance (Sp2) was used to calculate the sample size. The data were imported into PASS 11.0 software and the result was N3 = N4 = 11.

### Statistical analysis

All data were analysed using SPSS version 21.0. Continuous variables were summarised and expressed as mean ± standard deviation (SD) or median with 25%–75% interquartile range (IQR), depending on whether they fit a normal distribution. For data with a normal distribution, an unpaired t-test was used to assess the significance between the two groups. For data with a skewed distribution, the Mann–Whitney test was used. Categorical variables were expressed as frequencies (%) and compared using the chi-square test. Fisher’s exact test was used when the expected frequency < 5. Statistical significance was set at P < 0.05.

### Ethics approval

This study was approved by the Ethics Committee of Zhujiang Hospital of Southern Medical University and applied for exemption of informed consent (2023-KY-031-01). Our research complies with the provisions of the Declaration of Helsinki (revised 2013), and the registration number of Chinese Clinical Trial Registry is ChiCTR2300075901.

## Results

### Basic patient data and characteristics

**Figure 1:**
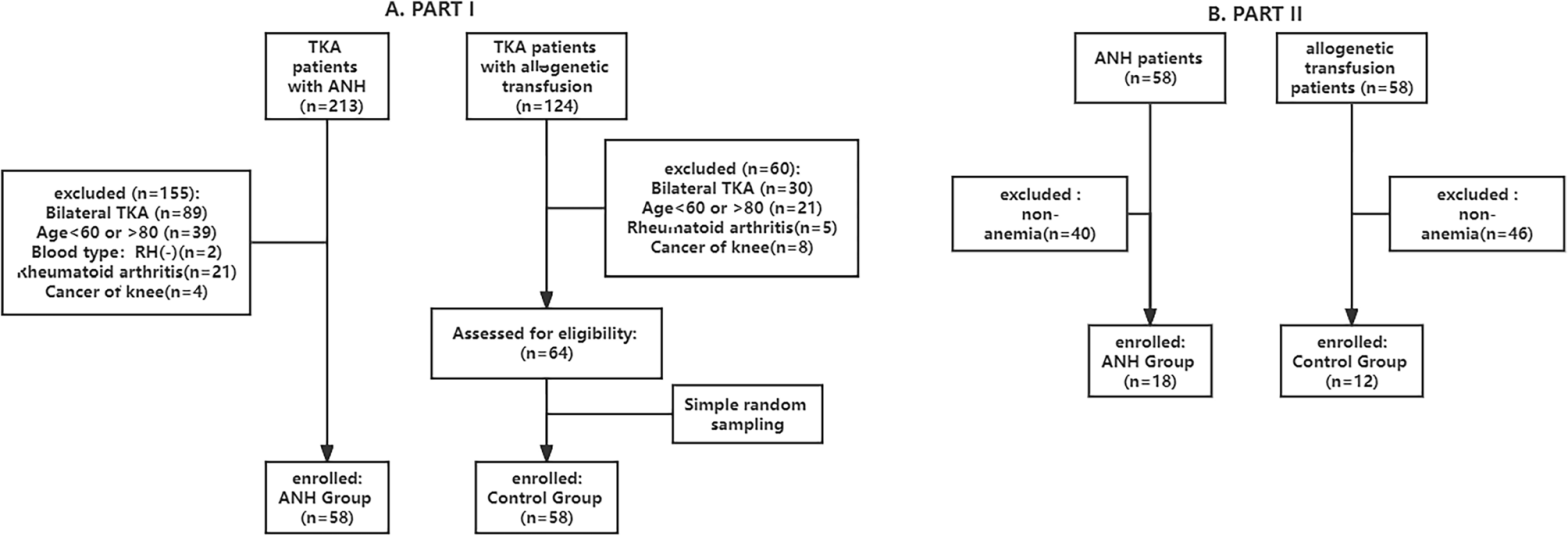
Screening process. (**A**) Screening out ANH and allogeneic transfusion medical records according to inclusion and exclusion criteria. (**B**) Screening out anemia medical records.

PART I: A total of 213 medical records of TKA using ANH were screened: 155 were excluded, and 58 composed the ANH group (n = 58). A total of 124 medical records of TKA surgery with allogeneic transfusion were screened again: 60 were excluded, and the researcher used simple random sampling from the 64 medical records, and 58 medical records were selected as the control group (n = 58), as shown in Fig. 1A.

PART II: According to the WHO definition of anaemia [1. Hb (adult male) < 130 g/L; 2. Hb (nonpregnant adult female) < 120 g/L], patients with anaemia were screened from part I of patients and divided into the ANH group (n = 18) and the control group (n = 12) according to whether ANH was used (Fig. 1B).

The two groups of PART I were matched for baseline demographics, comorbidities, and test results (Table 1).

**Table 1:** Comparison of preoperative data and test results.

	ANH?		P
	Yes(n = 58)	No(n = 58)	
Sex(male/fmale)	15/43	15/43	1.000
Weight (kg, x ± s)	60.24 ± 5.128	60.33 ± 5.514	0.931
Height (m, x ± s)	1.62 ± 0.061	1.62 ± 0.070	0.789
Pre-Hb (g/L, x ± s)	126.66 ± 13.388	131.41 ± 14.176	0.078
Pre-Hct (L/L, x ± s)	0.392 ± 0.036	0.404 ± 0.037	0.074
Pre-Plt (G/L, x ± s)	266.00 ± 65.12	269.33 ± 74.256	0.798
Pre-WBC (G/L, x ± s)	6.37 ± 1.643	6.99 ± 1.739	0.052
Pre-HSCRP (mg/L, x ± s)	2.21 ± 2.261	4.162 ± 7.827	0.072
Pre-ESR-T (mm/h, x ± s)	30.77 ± 16.696	35.65 ± 25.354	0.223
Pre-Urea (mmol/L, x ± s)	5.33 ± 1.381	5.47 ± 1.394	0.590
Pre-Cr (μmol/L, x ± s)	64.47 ± 16.152	69.96 ± 22.252	0.131
Pre-eGFR(x ± s)	86.69 ± 14.474	81.902 ± 15.786	0.091
Pre-CysC (mg/L, x ± s)	1.070 ± 0.239	1.139 ± 0.202	0.108
Pre-GGT (IU/L, x ± s)	28.58 ± 17.116	23.76 ± 9.441	0.117
Pre-ALT (IU/L, x ± s)	19.76 ± 12.22	16.91 ± 7.037	0.129
Pre-AST (IU/L, x ± s)	19.00 ± 7.255	18.00 ± 4.995	0.393
Pre-Glu (mmol/L, x ± s)	5.63 ± 1.375	5.61 ± 1.214	0.955
Pre-Alb (g/L, x ± s)	39.53 ± 3.58	40.82 ± 5.223	0.125
Pre-RPP(x ± s)	9308 ± 1870	9353 ± 2473	0.913
Pre-DP (mmHg, x ± s)	68.74 ± 10.740	69.67 ± 9.526	0.666
Pre-VAS(x ± s)	7.12 ± 1.044	7.22 ± 0.859	0.561
Operative time (min, x ± s)	61 ± 12.2	62 ± 13.6	1.000

Data are presented as the mean ± standard deviation (SD) and compared by one-way analysis of variance (one-way ANOVA) (*p < 0.05).

*Hb* hemoglobin, *Hct* hematocrit, *Plt* platelet count, *WBC* white blood cell, *HSCRP* high sensitivity C-reactive protein, *ESR-T* erythrocyte sedimentation rate, *Cr* creatinine, *eGFR* estimated glomerular filtration rate, *CysC* cystatin C, *GGT* glutamyl transferase, *ALT* alanine aminotransferase, *AST* aspartate aminotransferase, *Glu* glucose, *Alb* albumin, *OSMO* osmotic pressure, *RPP* rate-pressure-product, *DP* diastolic pressure, *VAS* visual analogue scale pain score.

The two groups of PART II were matched for baseline demographics, comorbidities, and test results (Supplementary Table S1).

Comparison of postoperative indicators between each group (Tables 2, 3, Supplementary Table S2).

**Table 2:** Comparison of postoperative indexes and their changes.

	ANH?		P
	YES(n = 58)	NO(n = 58)	
Post-Hb (g/L, x ± s)	108.16 ± 13.388	111.00 ± 14.176	0.271
Δ Hb (g/L, x ± s)	18.71 ± 8.840	20.41 ± 11.777	0.379
Theoretical loss of RBC (L, x ± s)	0.218 ± 0.104	0.241 ± 0.154	0.34
Post-Plt (G/L, x ± s)	221.69 ± 66.540	226.17 ± 69.295	0.723
Δ Plt (G/L, x ± s)	44.31 ± 37.757	43.16 ± 34.895	0.859
Post-WBC (G/L, x ± s)	10.22 ± 3.217	11.60 ± 3.017	0.019*
Post-HSCRP (mg/L, x ± s)	28.12 ± 27.249	39.53 ± 32.591	0.043*
Post-ESR-T (mm/h, x ± s)	34.931 ± 23.947	44.10 ± 34.368	0.098
Post-Urea (mmol/L, x ± s)	5.58 ± 1.754	6.43 ± 2.257	0.026*
Δ Urea (mmol/L, x ± s)	0.254 ± 1.759	0.960 ± 1.992	0.046*
Post-Cr (μmol/L, x ± s)	62.80 ± 16.613	68.24 ± 23.555	0.153
Δ Cr (μmol/L, x ± s)	− 1.66 ± 8.240	− 1.71 ± 9.542	0.978
Post-eGFR (x ± s)	88.05 ± 15.277	83.816 ± 15.882	0.146
Δ eGFR (x ± s)	1.35 ± 6.740	1.91 ± 7.390	0.673
Post-CysC (mg/L, x ± s)	0.95 ± 0.219	1.02 ± 0.223	0.130
Δ CysC (mg/L, x ± s)	0.112 ± 0.111	0.116 ± 0.158	0.881
post-GGT (IU/L, x ± s)	24.14 ± 13.390	22.29 ± 15.721	0.268
ΔGGT (IU/L, x ± s)	− 4.43 ± 6.387	− 1.47 ± 12.434	0.280
post-ALT (IU/L, x ± s)	17.53 ± 10.356	16.57 ± 9.891	0.616
ΔALT (IU/L, x ± s)	− 2.24 ± 7.820	− 0.34 ± 7.383	0.189
post-AST (IU/L, x ± s)	17.51 ± 5.192	18.74 ± 7.496	0.310
Δ AST (IU/L, x ± s)	− 1.49 ± 5.245	0.74 ± 5.866	0.035*
post-Glu (mmol/L, x ± s)	6.60 ± 2.049	6.51 ± 2.181	0.828
Δ Glu (mmol/L, x ± s)	0.971 ± 1.879	0.899 ± 1.972	0.841
post-Alb (g/L, x ± s)	34.04 ± 3.112	33.64 ± 3.632	0.527
Δ Alb (g/L, x ± s)	− 5.49 ± 2.627	− 7.17 ± 4.670	0.019*
post-RPP (x ± s)	9740 ± 1888	9764 ± 1840	0.945
post-DP (mmHg, x ± s)	66.41 ± 9.272	65.59 ± 9.526	0.636
D3-VAS (x ± s)	7.069 ± 0.934	7.069 ± 1.057	1.000
D7-VAS (x ± s)	2.000 ± 1.242	1.879 ± 1.257	0.604
M1-VAS (x ± s)	0.569 ± 0.728	0.552 ± 0.902	0.910
D3-ROM (x ± s)	75.17 ± 8.216	77.50 ± 8.946	0.147
M1-ROM (x ± s)	105.86 ± 8.279	106.12 ± 8.534	0.869
D7-WOMAC (x ± s)	5.89 ± 1.672	6.34 ± 1.628	0.146
M1-WOMAC (x ± s)	1.15 ± 1.056	1.15 ± 0.969	1.000

Data are presented as the mean ± standard deviation (SD) and compared by one-way analysis of variance (one-way ANOVA) (*p < 0.05).

*ROM* range of motion rom, *WOMAC* the Western Ontario and McMaster Universities osteoarthritis index, *D3* the 3rd day, *D7* the 7th day, *M1* the 1st month.

**Table 3:** Anemia*ANH effectiveness cross tabulation.

		If ANH is effective?		Sum	P
		Ineffective	Effective		
Anemia?	Normal	19	21	40	0.04
	Anemia	3	15	18	
Sum		22	36	58	

### Comparison of the degree of blood loss

No significant difference was noted in the postoperative Hb and Hb reduction between the ANH group and the control group (p > 0.05); no significant difference was observed in the theoretical loss of red blood cells between the two groups (P > 0.05); no significant postoperative difference in Plt and Plt reduction values were found (p > 0.05, Table 2).

ANH is more effective in treating patients with anaemia (p < 0.05, Table 3).

In patients with anaemia, the theoretical loss of red blood cells in the ANH group was significantly lower than that in the control group (p < 0.05); the two groups did not display significant difference in postoperative Plt and Plt reduction between patients (p > 0.05, Supplementary Table S2).

### Comparison of inflammatory indexes

The postoperative WBC count in the ANH group was significantly lower than that in the control group (p = 0.019 < 0.05); no significant difference was observed in the postoperative ESR-T between the two groups (p > 0.05); the postoperative HSCRP level in the ANH group was significantly lower than that in the control group (p = 0.043 < 0.05, Table 2).

In patients with anaemia, no significant difference was observed in the postoperative WBC count and HSCRP between the two groups (p > 0.05); the postoperative ESR-T level in the ANH group was significantly lower than that in the control group (p = 0.046 < 0.05, Supplementary Table S2).

### Comparison of renal function indexes

Compared with the control group, the postoperative serum urea in the ANH group was significantly lower (p = 0.026 < 0.05), and its elevated value was significantly less (p = 0.046 < 0.05). No significant differences in postoperative e-GFR and e-GFR changes, postoperative Cys-C and Cys-C reduction were noted (p > 0.05). No significant difference was observed in Urea, Cr, e-GFR and Cys-C between the two groups of patients with abnormal preoperative renal function indicators before and after surgery (p > 0.05, Table 2).

In patients with anaemia, no significant difference was observed in postoperative serum urea between the ANH group and the control group (P > 0.05); no significant difference was noted in the postoperative Cr and Cr reduction between the two groups (p > 0.05). No significant difference was observed in the postoperative e-GFR and e-GFR changes (p > 0.05); no significant difference was noted in the postoperative Cys-C and Cys-C reduction between the two groups (p > 0.05, Supplementary Table S2).

In patients with renal dysfunction, all indicators were close (p > 0.05) except Hb and Hct (p < 0.05), and ANH was more effective in patients with anaemia (p < 0.05), as shown in Supplementary Table S3A,B.

### Comparison of liver function indexes

No significant difference was observed in the postoperative ALT and ALT changes, postoperative GGT and GGT changes, and postoperative AST between the ANH group and the control group (p > 0.05). Amongst the two groups of patients with abnormal preoperative liver function indicators, the preoperative GGT in the ANH group was significantly higher than that in the control group (p < 0.05), and the other indicators had no significant difference (p > 0.05). No significant difference (p > 0.05) was found, and no significant difference was observed in GGT changes (p > 0.05). The changes of ALT and AST in the ANH group decreased, and a significant difference compared with the control group was noted (p < 0.05), as shown in Table 2.

In patients with anaemia, no significant difference was observed in the postoperative GGT and GGT changes between the ANH group and the control group (p > 0.05); no significant difference was noted in the postoperative ALT and ALT changes between the two groups (p > 0.05); no significant difference was found in the postoperative AST and AST changes between the two groups (p > 0.05), as shown in Supplementary Table S2.

In patients with abnormal liver function, all indicators were close (p > 0.05, Supplementary Table S3C).

### Comparison of nutritional status indicators in each group of patients

No significant difference was noted in the postoperative Glu and Glu changes between the ANH group and the control group (p > 0.05); no significant difference was observed in the postoperative serum Alb between the two groups (p > 0.05); the postoperative serum Alb in the ANH group was significantly lower than that in the control group (p = 0.019 < 0.05, Table 2).

In patients with anaemia, no significant difference was observed in the postoperative Glu and Glu changes between the ANH group and the control group (p > 0.05); no significant difference was noted in the postoperative serum Alb and its decreasing value between the two groups (p > 0.05), as shown in Supplementary Table S2.

### Comparison of cardiac function indexes in each group of patients

No significant difference was observed between the ANH group and the control group in the postoperative heart rate systolic blood pressure product and diastolic blood pressure (p > 0.05). No significant difference was noted in the preoperative and postoperative heart rate and systolic blood pressure product and its changes in the two groups of patients with preoperative myocardial ischemia (p > 0.05, Table 2).

In patients with anaemia, no significant difference was observed in the postoperative heart rate systolic blood pressure product and diastolic blood pressure between the ANH group and the control group (p > 0.05, Supplementary Table S2).

In patients with myocardial ischemia, all indicators were close (p > 0.05, Supplementary Table S3D).

### Comparison of perioperative pain and knee function in each group

No significant difference was observed in the VAS score, knee ROM and WOMAC knee score between the ANH group and the control group at various time points after the operation (p > 0.05, Table 2).

In patients with anaemia, no significant difference was noted in the VAS score, knee ROM and WOMAC knee score between the two groups at different time points after the operation (p > 0.05, Supplementary Table S2).

## Discussion

**Figure 2:**
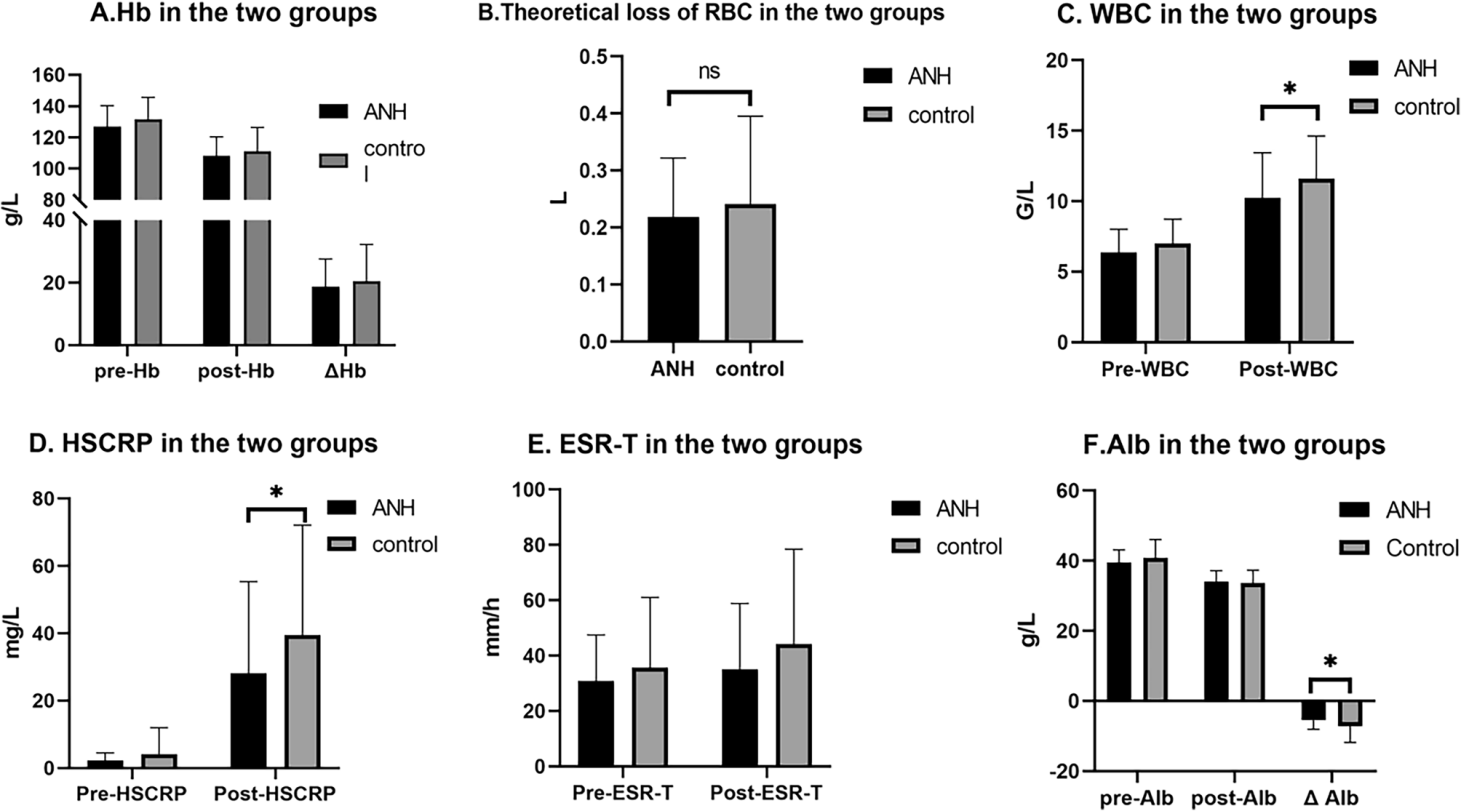
The effectiveness of ANH in TKA. (**A**,**B**) ANH have the same perioperative hematological indexes as allogeneic transfusion. (**C**–**F**) ANH have the lower perioperative inflammatory indexes than allogeneic transfusion (*P < 0.05).

**Figure 3:**
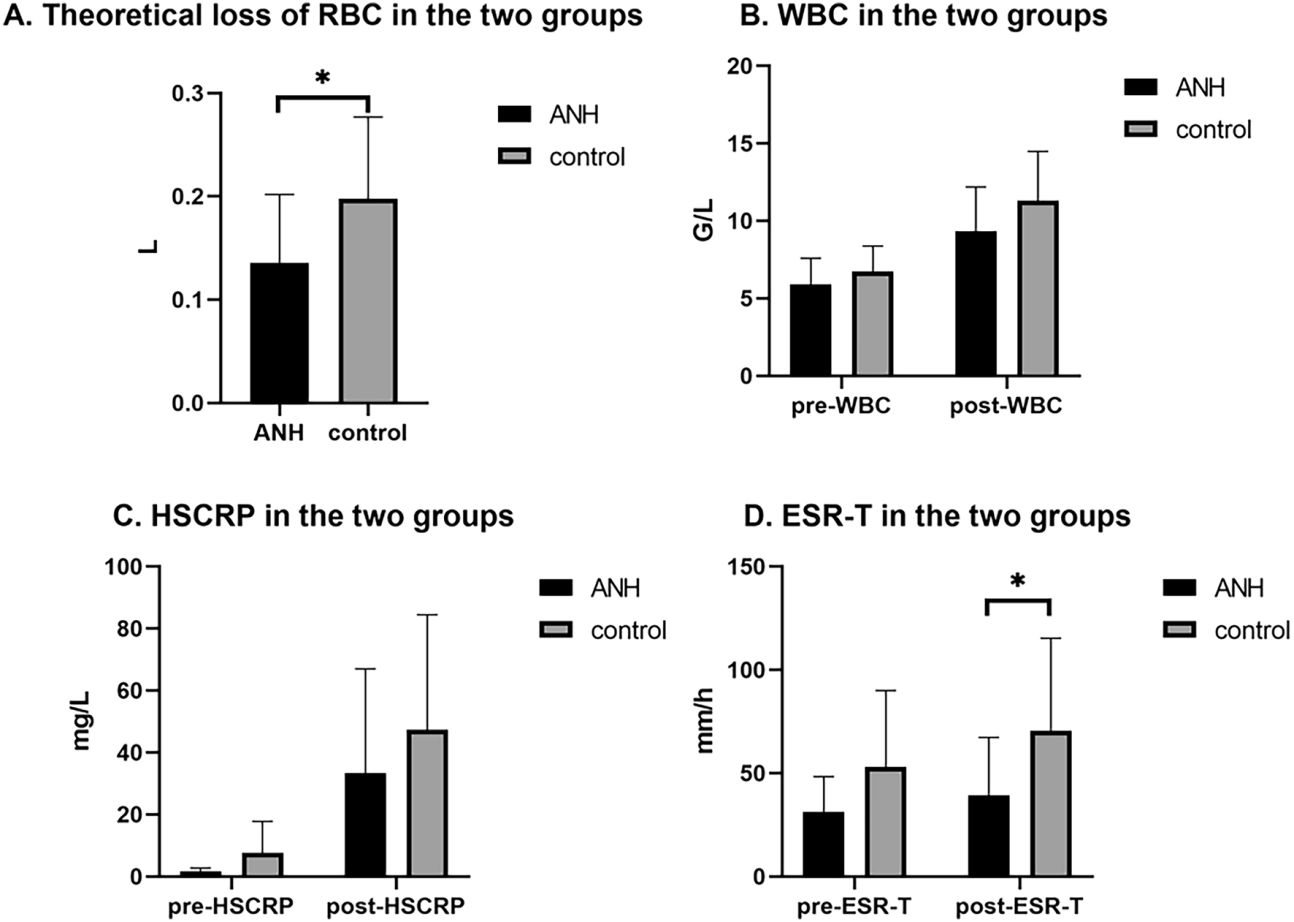
The effectiveness of ANH in TKA of anemia patients. (**A**) ANH have the better perioperative hematological indexes than allogeneic transfusion. (**B**–**D**) ANH have the lower perioperative inflammatory indexes than allogeneic transfusion. (*P < 0.05).

The results verified that ANH had the same perioperative haemoprotective effect as allogeneic transfusion in patients undergoing TKA. For patients with anaemia, the perioperative blood protection effect of ANH was significantly better than that of patients without anaemia (Figs. 2 and 3).

The perioperative blood management of patients who underwent TKA also made some progress, including the preoperative, intraoperative and postoperative scheme (preoperative anaemia correction, preoperative autologous blood donation, intraoperative use of tranexamic acid and other drugs, intraoperative use of pneumatic tourniquet, intraoperative acute normovolemic haemodilution, intraoperative red blood cell recovery, postoperative drainage pipe clamp, postoperative anaemia correction and other procedures)^24^. With the implementation of numerous perioperative blood management programmes and the support of highly skilled surgical techniques, the role of ANH was not as great as expected. However, ANH is still an alternative measure to allogeneic transfusion.

However, for patients with anaemia, the effectiveness of ANH was significantly better than that of normal patients considering several aspects: ① Due to the anaemic state and TKA surgical trauma, the patient’s extramedullary hematopoietic system was activated, which in turn increased the patient’s red blood cells. Under the same conditions of surgical trauma, the activation of the extramedullary hematopoietic system was higher in patients with anaemia. ② Patients with anaemia have fewer red blood cells than normal patients. When the same amount of blood was drawn when taking ANH, this accounted for a larger proportion of blood volume in patients with anaemia than in normal patients, and the final return of autologous blood resulted in better results. ③ Compared with normal patients, the Hct in patients with anaemia was closer to the critical value of ANH production after ANH application. Therefore, anaemia can be considered for inclusion in the indication of ANH.

### ANH can significantly reduce postoperative inflammation

The stress of TKA surgical trauma is the activation of neutrophils and vascular endothelial cells through the neuroendocrine system and immune system, and the release of inflammatory mediators such as tumour necrosis factor TNF-α, interleukin IL-6, oxygen free radicals and interleukin IL-1β simultaneously producing other molecules that affect endothelial cells^25^ activate the body’s cytokine network, leading to the proliferation and release of inflammatory cells. Tumour necrosis factor TNF-α has a wide range of biological effects and is a cytokine that activates the body’s inflammatory response. In the early stage of inflammation, TNF-α activates the body’s immune system to produce and release secondary inflammatory mediators; interleukin IL-6 is a pro-inflammatory cytokine that participates in the body’s inflammatory response and plays a key role. It indicates the initiation of the body’s inflammatory response and the persistent state of the inflammatory response; HSCRP is an acute inflammatory response. The reactive protein produced during IL-26 is mainly regulated and mediated by IL-26 and is generated and released in the liver. It mainly activates complement, promotes phagocytosis and participates in the body’s inflammatory response. It has a certain immune function. Regulation. In the case of injury, infection, tissue inflammation, or necrosis, the level of HSCRP increases rapidly. When the disease subsides, the level of HSCRP rapidly drops to the normal level. It is less affected by other factors and is an accurate, sensitive indicator of local/systemic infection in the body.

The use of pneumatic tourniquets also affects inflammatory factors in the body. On the one hand, during the use of the pneumatic tourniquet, the limbs are affected by ischemia and hypoxia; on the other hand, after the pneumatic tourniquet is removed, the limbs react with hyperaemia, so that the blood flow in the dermis of the limbs can reach the normal value 4 times highercausing local swelling and compression and massive perfusion of inflammatory mediators; causing the body’s inflammatory response and the possibility of postoperative complications^26,27^.

In this paper, under the stimulation of TKA surgery, anaesthesia and pneumatic tourniquet, the postoperative inflammatory index of the ANH group compared with the control group was significantly lower, indicating the postoperative inflammatory response in the ANH group was significantly lighter than that in the control group. The application of ANH in patients with anaemia can also reduce the postoperative inflammatory response. The reasons were mainly considered in the following aspects: ① ANH returned autologous blood, which improved the partial pressure of oxygen in tissues and organs in the body, alleviated reperfusion injury caused by surgery, anaesthesia and tourniquet stimulation, and inhibited the release of inflammatory cytokines^28^. ② AHH increased the body’s postoperative blood volume and directly diluted the body’s blood circulation cortisol concentration and the plasma level of catecholamines, which was important for reducing the body’s stress response. ③ Kahvegian and Öztürk’s studies have shown that ANH can activate immune cells and increase the production and release of inflammatory factors; this will not cause a strong immune response in the body but will enhance the body’s immunity and reduce the risk of infection^29,30^ (Figs. 2 and 3).

### Renal safety of ANH

Combined with various indicators and their changes, the results reveal ANH neither damages the renal function nor causes a greater burden to patients with abnormal preoperative renal function indicators. The reason for the increase of serum urea should be considered during the perioperative period of fasting, fever or vomiting, which leads to dehydration symptoms in the patient’s body, which leads to the increase of serum urea. ANH supplements the effective blood volume and is effective in relieving the symptoms of dehydration in the body, so the elevated value of serum urea was significantly lower than that of the control group.

Studies have shown that the kidney is the organ most vulnerable to hypoxia-induced damage when haemodilution is performed; in an animal model, van Bommel et al. observed an early limitation of microvascular oxygenation on the renal surface during progressive hemodilution^31^. In humans, renal and visceral injury can be observed in patients with low Hb concentrations during CPB^32^. Clinical studies have demonstrated for the first time that renal anaemia is poorly tolerated, suggesting that haemodilution and concomitant low Hb levels are independent risk factors for acute renal failure after cardiac surgery^33^. In a mouse haemodilution model, Van Bommel et al. observed a decrease in microvascular renal pO2 immediately after the onset of haemodilution despite an initial increase in renal blood flow^34^. In addition, ANH enables the kidney to compensate for hypoxia–ischemia by improving micro rental circulatory perfusion^35^. Crystal^36^ pointed out that when haemodilution was performed, when Hct = 30%, the renal blood oxygen delivery began to decrease, and when Hct = 10%, the renal blood oxygen delivery was only 1/4 of the baseline value. The effect of mild ANH on the kidney was still within its compensatory range, and mild ANH will not damage renal function (Supplementary Figs. S1 and S2).

### Hepatic safety of ANH

Combined with various indicators and their changes, the results reveal that ANH does not damage the liver function of patients with TKA, and the application of ANH in patients with anaemia is even beneficial to the improvement of liver function. The liver function of patients who undergo ANH is still in normal compensation and not damaged. Even anaemia, surgical stress and inflammation trigger the patient’s extramedullary hematopoietic system, including the liver, resulting in lower GGT, ALT and AST in patients with anaemia than in patients with normal Hb (Supplementary Figs. S1 and S2).

Amongst the organs of the body, the liver has strong anti-ischemia and hypoxia properties, and its abilities to compensate for hypoxia and ischemia are extremely strong. This conclusion is consistent with other animal models^37^ and human studies^34^ showing the liver is uniquely tolerant to dilutional anaemia. In a progressive isovolumic haemodilution model, Nielsen et al.^38^ observed no change in the serum lactate concentration, arterial ketone body ratio or liver histological damage until the Hct dropped to 8%. Thus the threshold value of liver tolerance to ANH has not yet been determined, and certainly, mild ANH will not damage the liver^39^.

### Myocardial safety of ANH

RPP is the double product of heart rate and systolic blood pressure and is an effective indicator to reflect myocardial oxygen consumption. RPP is widely used in modern medicine because of its simple calculation and convenient measurement. Elevated RPP is associated with the occurrence of adverse cardiovascular events and myocardial ischemia perfusion injury, which suggests coronary insufficiency. The domestic research on RPP and myocardial ischemia can conclude that compared with the simple heart rate, the RPP has better diagnostic significance as the threshold of myocardial ischemia^40^. RPP reflects the subjects’ cardiovascular status and myocardial hypoxia–ischemia. When RPP suddenly rises to a certain level (12,000), it reflects the possibility of hypoxic ischemia in the myocardium^41^. RPP is correlated with cardiovascular diseases such as myocardial ischemia, arterial occlusion, arterial plaque, arteriosclerosis and pregnancy eclampsia. RPP can predict the changes and prognosis of patients with heart failure and facilitate clinical diagnosis.

In this trial, no significant difference was observed in the postoperative RPP and DP of the patients in each group (p > 0.05). The RPP of all patients increased after the operation, and no significant difference was noted in the increase between the two groups. Under the influence of factors such as anaesthesia, surgery and injury, RPP increased, reflecting the increase in myocardial oxygen consumption, which was still within the normal range. However, ANH did not have an additional effect on the rise in RPP. Considering that the Hb concentration decreased after ANH, the oxygen-carrying capacity of the same amount of blood decreased, but the body strengthened the myocardial function, increased the cardiac output and frequency, and haemodilution reduced blood viscosity, improved microcirculation and improved tissue. Which compensate for oxygen uptake and utilisation and other mechanisms, and maintain the oxygen supply and utilisation of body tissues. Studies have shown that with a significant decrease in Hct and Hb concentrations, the body’s cardiac index increases, and the body compensates in this way to keep tissue oxygen supply stable (Fig. 2 and Supplementary Fig. S2).

The effects of ANH on the heart and myocardium of the body include the following: ① Myocardial functional capacity: Hofmann–Kiefer et al. observed that with blood dilution, the resistance of peripheral blood vessels decreases, and cardiac output and myocardial contractility increase significantly. However, the parameters representing myocardial hypoxic–ischemic load and pulmonary vascular permeability remains unchanged. Thus, the application of ANH improves the ability of the myocardium to perform work^42^. ② Myocardial oxygen supply and demand: Zhang Liang’s research confirmed 6% hydroxyethyl starch ANH had no significant effect on the internal environment and myocardial oxygen metabolism of patients undergoing off-pump cardiac surgery; the oxygen-carrying capacity of the same amount of blood decreased, and the body improved the oxygen-uptake capacity of myocardial cells, reduced the work of myocardial beating, reduced the oxygen consumption required by myocardial cells and balanced the oxygen supply and oxygen consumption of the myocardium^43^. Myocardial cells can compensate for the hypoxic–ischemic state caused by mild ANH and will not cause additional damage to the myocardium. The blood viscosity improved the muscle strength required for the work of the myocardial movement.

### ANH can reduce the loss of Alb

Alb is the most abundant plasma protein in the human body. Alb is an indicator commonly used to evaluate the nutritional status of the body and participates in the body’s inflammatory immune response. Under the regulation of insulin and glucagon, the decomposition of Glu in normal patients is closely related to the inflammatory immune response of the body. The synthesis is in dynamic equilibrium, and the serum Glu level is generally in a dynamic and stable state; the plasma osmotic pressure is mainly generated by osmotic active substances, including Na ions, Alb and other substances, which mainly play a role in maintaining the homeostasis of the internal environment, and the level of osmotic pressure also reflects the smooth operation of the body fluid regulation system^44^.

In this experiment, the main consideration is that surgical trauma stress leads to an increase in the synthesis and secretion of epinephrine and norepinephrine, and sympathetic nerve stimulation stimulates islet A cells to secrete high Glu, which inhibits insulin secretion, leading to postoperative Glu increased stress^45^. In all patients, no significant difference was observed in the decrease of Alb between the ANH group and the control group (p = 0.055); amongst patients with ANH, the decrease of Alb was lower in patients with anaemia; amongst patients with anaemia, the Alb decrease was lower in patients with ANH. The reason is that Alb is lost in the perioperative period with haemorrhage during the perioperative period, whilst ANH reduces the loss of Alb in the perioperative period due to haemodilution before the operation, the loss of Alb is reduced and the self-transfusion of autologous blood contains Alb after the operation. Combined with the above-mentioned Alb involved in the body’s inflammatory immune response, the application of ANH has a positive effect on enhancing the body’s immunity (Fig. 2).

### ANH does not affect postoperative pain and knee function

In this trial, no significant difference was observed in the VAS score, knee ROM and WOMAC knee score between the groups of patients at each time point after the operation. Considering that ANH mainly affected blood circulation, it had no influence on local muscles and nerves of the knee joint and may affect the blood supply around the knee joint, including the blood supply of the quadriceps muscle, but this effect was too small and not reflected in the patient’s postoperative examination and rehabilitation.

### Deficiencies and prospects

Some limitations of the study may affect the generalisability of the findings. One limitation is the retrospective design of the study, which may introduce bias and limit the ability to establish causality. Another limitation is the small sample size, which may affect the statistical power of the study and limit the generalisability of the findings to other populations. Additionally, the study was conducted at a single centre, which may limit the generalisability of the findings to other settings. In addition, coagulation function is also an important point in ANH evaluation. Coagulation profile, INR, FDPs and other indicators were understudied in this study. Therefore, a related multicentre prospective clinical study is being conducted, which we hope will be beneficial to the blood management of patients.

## Conclusion

ANH has the same perioperative haemoprotective effect as allogeneic transfusion in TKA and reduces postoperative inflammatory reaction. For patients with anaemia, the perioperative blood protection effect of TKA patients with ANH is significantly better than that of patients without anaemia; it indicates the selection of ANH in TKA. In other aspects (such as renal function, liver function, renal function and other indicators), the application of ANH in patients also has certain advantages. In general, under the premise of safe, standardised operation, ANH is beneficial for patients, and the body’s tolerance to ANH is within the body’s compensation range.

### Supplementary Information

Supplementary Information.

## Data Availability

The data used to support the outcome of the study are available from the corresponding author upon reasonable request.
