# Supplementary material for: Effectiveness, safety and indications of acute normovolemic haemodilution in total knee arthroplasty
Source: Sci Rep. 2024 Feb 8;14:3298. doi: 10.1038/s41598-024-53779-6 (PMC10853272; doi:10.1038/s41598-024-53779-6)
Supplement: Supplementary file 1 — Supplementary Information. [file 41598_2024_53779_MOESM1_ESM.docx]

**The Effectiveness, Safety, and Indications of Acute Normovolemic Hemodilution in**

**Total Knee Arthroplasty**

Yucong Li^1†^,Jingle Chen^1†^, Hao Xie^1†^, Hangxing Wu^1^, Zhijie Zuo^1^, wanyan Hu^1^, Chao Xie^1^*, Lijun Lin^1^*

^1^ Department of joint and orthopedic, Zhujiang Hospital, Southern Medical University, GuanZhou, 510220, P. R. China

[^*^ Corresponding author: chaoxie1118@foxmail.com ; gost1@smu.edu.cn](mailto:*%20Corresponding%20author:%20chaoxie1118@foxmail.com%20;%20gost1@smu.edu.cn)

^#^ These authors contributed equally to this work.

Table S1. Comparison of preoperative data and test results of anemia patients

|  | ANH? | | P |
| --- | --- | --- | --- |
|  | YES(n=18) | NO(n=12) |  |
| Sex(男/女) | 7/11 | 3/9 | 0.438 |
| Weight(kg, x±s) | 59.77±5.461 | 59.91±3.987 | 0.936 |
| Height(m, x±s) | 1.62±0.062 | 1.61±0.063 | 0.780 |
| Pre-Hb(g/L, x±s) | 111.78±10.29 | 113.67±8.46 | 0.588 |
| Pre-Hct(L/L, x±s) | 0.352±0.026 | 0.360±0.024 | 0.417 |
| Pre-Plt(G/L, x±s) | 266.39±77.254 | 309.33±120.944 | 0.291 |
| Pre-WBC(G/L, x±s) | 5.91±1.677 | 6.74±1.637 | 0.187 |
| Pre-HSCRP(mg/L, x±s) | 1.66±1.153 | 7.59±10.234 | 0.070 |
| Pre-ESR-T(mm/h, x±s) | 31.28±17.001 | 53.08±37.042 | 0.077 |
| Pre-Urea(mmol/L, x±s) | 4.94±1.109 | 5.23±1.336 | 0.540 |
| Pre-Cr(μmol/L, x±s) | 64.12±18.545 | 63.12±23.902 | 0.901 |
| Pre-eGFR(x±s) | 89.18±11.704 | 86.91±15.313 | 0.668 |
| Pre-CysC(mg/L, x±s) | 1.03±0.192 | 1.13±0.241 | 0.244 |
| Pre-GGT(IU/L, x±s) | 21.29±7.295 | 23.08±11.373 | 0.637 |
| Pre-ALT(IU/L, x±s) | 12.12±3.951 | 15.50±8.006 | 0.197 |
| Pre-AST(IU/L, x±s) | 14.65±2.936 | 18.00±5.592 | 0.076 |
| Pre-Glu(mmol/L, x±s) | 5.51±1.794 | 5.35±1.324 | 0.782 |
| Pre-Alb(g/L, x±s) | 38.69±3.205 | 37.42±3.376 | 0.314 |
| Pre-RPP(x±s) | 9271±2284 | 10485±2873 | 0.234 |
| Pre-DP(mmHg, x±s) | 68.28±11.018 | 68.08±13.886 | 0.968 |
| Pre-VAS(x±s) | 7.22±1.309 | 7.42±0.793 | 0.617 |

Data are presented as the mean±standard deviation (SD) and compared by one-way analysis of variance (one-way ANOVA).(*: p＜0.05)

Table S2. Comparison of postoperative indexes and their changes in anemic patients

|  | ANH? | | P |
| --- | --- | --- | --- |
|  | YES（n=18） | NO（n=12） |  |
| Theoretical loss of RBC（L，x±s） | 0.136±0.066 | 0.198±0.079 | 0.034* |
| Post- Plt（G/L，x±s） | 222.05±83.605 | 279.75±109.83 | 0.138 |
| Variation in Plt （G/L，x±s） | -44.33±39.519 | -29.58±28.915 | 0.248 |
| Post- WBC（G/L，x±s） | 9.32±2.873 | 11.31±3.181 | 0.095 |
| Post- HSCRP（mg/L，x±s） | 33.278±33.801 | 47.38±37.071 | 0.303 |
| Post- ESR-T（mm/h，x±s） | 39.16±28.197 | 70.50±44.817 | 0.046* |
| Post- Urea（mmol/L，x±s） | 5.20±1.832 | 6.13±1.691 | 0.166 |
| Variation in Urea（mmol/L，x±s） | 0.256±1.855 | 0.896±1.689 | 0.337 |
| Post- Cr（μmol/L，x±s） | 63.28±21.380 | 62.30±23.902 | 0.803 |
| Variation in Cr（μmol/L，x±s） | -0.83±8.183 | -1.82±11.501 | 0.800 |
| Post- eGFR（x±s） | 90.61±13.405 | 86.91±14.119 | 0.741 |
| Variation in eGFR（x±s） | 1.43±6.056 | 1.97±7.889 | 0.842 |
| Post- CysC（mg/L，x±s） | 0.92±0.224 | 1.03±0.269 | 0.274 |
| Variation in CysC（mg/L，x±s） | -0.11±0.079 | -0.10±0.224 | 0.923 |
| Post- GGT（IU/L，x±s） | 18.00±4.834 | 21.41±11.016 | 0.331 |
| Variation in GGT（IU/L，x±s） | -3.29±3.754 | -1.66±4.228 | 0.297 |
| Post- ALT（IU/L，x±s） | 11.76±3.211 | 14.58±5.775 | 0.145 |
| Variation in ALT（IU/L，x±s） | -0.35±3.239 | -0.91±4.187 | 0.700 |
| Post- AST（IU/L，x±s） | 15.23±3.307 | 16.83±2.855 | 0.177 |
| Variation in AST（IU/L，x±s） | -0.59±3.083 | -1.17±4.529 | 0.259 |
| Post- Glu（mmol/L，x±s） | 6.83±2.464 | 6.04±0.930 | 0.226 |
| Variation in Glu（mmol/L，x±s） | 1.32±2.429 | 0.68±1.060 | 0.336 |
| Post- Alb（g/L，x±s） | 34.26±3.139 | 32.15±3.783 | 0.124 |
| Variation in Alb（g/L，x±s） | -4.42±2.865 | -5.27±2.305 | 0.380 |
| Post- RPP（x±s） | 9459±1939 | 9882±1798 | 0.546 |
| Post- DP（mmHg，x±s） | 69.7±12.35 | 63.5±10.21 | 0.142 |
| D3-VAS（x±s） | 6.83±0.923 | 6.58±0.996 | 0.495 |
| D7-VAS（x±s） | 1.72±1.127 | 1.91±0.793 | 0.584 |
| M1-VAS（x±s） | 0.55±0.704 | 0.91±1.083 | 0.322 |
| D3-ROM（x±s） | 75.00±9.235 | 75.83±10.408 | 0.824 |
| M1-ROM （x±s） | 106.38±7.236 | 105.41±6.5566 | 0.706 |
| D7-WOMAC（x±s） | 5.61±1.501 | 6.00±1.954 | 0.566 |
| M1-WOMAC（x±s） | 1.05±1.259 | 1.08±0.996 | 0.947 |

Data are presented as the mean±standard deviation (SD) and compared by one-way analysis of variance (one-way ANOVA).

Table S3A. Comparison of test results in patients with renal dysfunction

|  | ANH? | | P |
| --- | --- | --- | --- |
|  | YES(n=39) | NO(n=44) |  |
| Sex(male/female) | 10/29 | 12/32 | 0.868 |
| Weight(kg, x±s) | 60.28±5.01 | 60.84±5.87 | 0.628 |
| Height(m, x±s) | 1.62±0.061 | 1.63±0.071 | 0.661 |
| Pre- Hb(g/L, x±s) | 125.45±13.93 | 133.11±15.26 | 0.018* |
| Pre- Hct(L/L, x±s) | 0.38±0.036 | 0.40±0.041 | 0.014* |
| Theoretical loss of RBC(L, x±s) | 0.205±0.099 | 0.262±0.162 | 0.050* |
| Pre- WBC(G/L, x±s) | 6.33±1.59 | 7.22±1.81 | 0.090 |
| Post- WBC(G/L, x±s) | 10.08±2.70 | 11.76±2.59 | 0.005* |
| Pre- HSCRP(mg/L, x±s) | 2.56±2.59 | 4.36±8.62 | 0.194 |
| Post- HSCRP(mg/L, x±s) | 30.20±28.61 | 40.08±34.78 | 0.195 |
| Pre- ESR-T(mm/h, x±s) | 33.2±16.84 | 36.8±26.31 | 0.446 |
| Post- ESR-T(mm/h, x±s) | 38.77±26.71 | 46.65±37.51 | 0.268 |
| Pre- Urea(mmol/L, x±s) | 5.36±1.51 | 5.72±1.42 | 0.540 |
| Post- Urea(mmol/L, x±s) | 5.78±1.92 | 6.51±2.48 | 0.135 |
| Pre- Cr(μmol/L, x±s) | 68.78±16.86 | 75.99±22.15 | 0.096 |
| Post- Cr(μmol/L, x±s) | 68.07±16.73 | 73.42±24.57 | 0.243 |
| Pre- eGFR(x±s) | 82.02±15.01 | 77.43±15.36 | 0.171 |
| Post- eGFR(x±s) | 82.85±15.50 | 79.90±15.94 | 0.393 |
| Pre- CysC(mg/L, x±s) | 1.16±0.21 | 1.20±0.175 | 0.375 |
| Post- CysC(mg/L, x±s) | 1.03±0.20 | 1.07±0.22 | 0.461 |
| Pre- GGT(IU/L, x±s) | 31.0±19.31 | 24.22±7.85 | 0.052 |
| Post- GGT(IU/L, x±s) | 25.54±14.9 | 21.13±8.1 | 0.114 |
| Pre- ALT(IU/L, x±s) | 20.13±13.15 | 16.63±6.89 | 0.147 |
| Post- ALT(IU/L, x±s) | 17.68±11.48 | 16.09±9.81 | 0.508 |
| Pre- AST(IU/L, x±s) | 19.39±8.40 | 18.20±4.93 | 0.446 |
| Post- AST(IU/L, x±s) | 17.86±5.96 | 18.95±7.83 | 0.479 |
| Pre- RPP(x±s) | 9104±1853 | 9055±2357 | 0.915 |
| Post- RPP(x±s) | 9524±1784 | 9613±1761 | 0.915 |
| Pre- DP(mmHg, x±s) | 68.77±11.24 | 68.98±12.47 | 0.945 |
| Post- DP(mmHg, x±s) | 67.25±10.30 | 65.86±9.61 | 0.822 |

Data are presented as the mean±standard deviation (SD) and compared by one-way analysis of variance (one-way ANOVA).(*: p＜0.05)

Table S3.B. anemia*ANH effectiveness cross tabulation

|  | | If ANH is effective？ | | Sum | P |
| --- | --- | --- | --- | --- | --- |
|  |  | Ineffective | Effective |  |  |
| Anemia? | Normal | 8 | 17 | 25 | 0.004* |
|  | Anemia | 11 | 3 | 24 |  |
| Sum | | 19 | 20 | 39 |  |

*: p＜0.05

Table S3.C. Comparison of test results in patients with abnormal liver function

|  | ANH? | | P |
| --- | --- | --- | --- |
|  | YES(n=16) | NO(n=14) |  |
| Sex(male/female) | 1/15, 7% | 2/12,13% | 0.468 |
| Weight(kg, x±s) | 60.28±5.01 | 60.84±5.87 | 0.628 |
| Height(m, x±s) | 1.62±0.061 | 1.63±0.071 | 0.661 |
| Pre- Hb(g/L, x±s) | 130.43±8.06 | 133.85±18.35 | 0.528 |
| Pre- Hct(L/L, x±s) | 0.40±0.019 | 0.41±0.046 | 0.481 |
| Theoretical loss of RBC(L, x±s) | 0.215±0.107 | 0.213±0.123 | 0.965 |
| Pre- WBC(G/L, x±s) | 7.35±1.85 | 7.87±2.48 | 0.528 |
| Post- WBC(G/L, x±s) | 11.08±2.86 | 11.73±2.81 | 0.538 |
| Pre- HSCRP(mg/L, x±s) | 3.10±2.84 | 9.05±14.53 | 0.154 |
| Post- HSCRP(mg/L, x±s) | 32.00±32.18 | 47.44±40.44 | 0.263 |
| Pre- ESR-T(mm/h, x±s) | 37.37±15.87 | 45.14±30.13 | 0.365 |
| Post- ESR-T(mm/h, x±s) | 38.43±22.58 | 48.14±34.02 | 0.374 |
| Pre- Urea(mmol/L, x±s) | 5.28±1.53 | 4.79±0.92 | 0.682 |
| Post- Urea(mmol/L, x±s) | 5.51±1.53 | 5.76±1.73 | 0.682 |
| Pre- Cr(μmol/L, x±s) | 64.87±19.39 | 73.27±21.85 | 0.279 |
| Post- Cr(μmol/L, x±s) | 61.73±16.61 | 70.42±23.37 | 0.258 |
| Pre- eGFR(x±s) | 82.48±19.77 | 79.24±15.89 | 0.615 |
| Post- eGFR(x±s) | 84.48±19.77 | 82.29±15.59 | 0.737 |
| Pre- CysC(mg/L, x±s) | 1.14±0.29 | 1.20±0.21 | 0.548 |
| Post- CysC(mg/L, x±s) | 1.01±0.27 | 1.06±0.18 | 0.535 |
| Pre- GGT(IU/L, x±s) | 48.37±18.50 | 36.64±6.60 | 0.078 |
| Post- GGT(IU/L, x±s) | 39.93±12.85 | 39.35±24.08 | 0.937 |
| Pre- ALT(IU/L, x±s) | 29.43±13.64 | 20.28±8.73 | 0.086 |
| Post- ALT(IU/L, x±s) | 24.25±13.72 | 24.78±15.85 | 0.922 |
| Pre- AST(IU/L, x±s) | 24.81±8.95 | 20.71±7.35 | 0.180 |
| Post- AST(IU/L, x±s) | 21.12±6.85 | 24.57±12.50 | 0.370 |
| Pre- RPP(x±s) | 9352±1706 | 10649±2501 | 0.116 |
| Post- RPP(x±s) | 10051±2228 | 10331±1983 | 0.718 |
| Pre- DP(mmHg, x±s) | 65.00±10.97 | 68.64±12.64 | 0.410 |
| Post- DP(mmHg, x±s) | 64.56±7.55 | 60.64±7.13 | 0.155 |

Data are presented as the mean±standard deviation (SD) and compared by one-way analysis of variance (one-way ANOVA).(*: p＜0.05)

Table S3.D. Comparison of test results in patients with myocardial ischemia

|  | ANH? | | P |
| --- | --- | --- | --- |
|  | YES(n=5) | NO(n=8) |  |
| Sex(male/female) | 2/3, 40% | 4/4,50% | 0.754 |
| Weight(kg, x±s) | 59.20±6.97 | 63.25±4.77 | 0.295 |
| Height(m, x±s) | 1.63±0.079 | 1.61±0.094 | 0.682 |
| Pre- Hb(g/L, x±s) | 129.80±17.49 | 122.12±13.14 | 0.428 |
| Pre- Hct(L/L, x±s) | 0.40±0.049 | 0.37±0.034 | 0.229 |
| Theoretical loss of RBC(L, x±s) | 0.257±0.105 | 0.172±0.110 | 0.201 |
| Pre- WBC(G/L, x±s) | 7.02±1.82 | 7.40±2.77 | 0.773 |
| Post- WBC(G/L, x±s) | 13.16±6.26 | 11.52±2.17 | 0.600 |
| Pre- HSCRP(mg/L, x±s) | 1.76±1.32 | 9.34±16.16 | 0.228 |
| Post- HSCRP(mg/L, x±s) | 26.42±15.43 | 27.43±22.24 | 0.925 |
| Pre- ESR-T(mm/h, x±s) | 17.2±6.72 | 36.1±25.99 | 0.084 |
| Post- ESR-T(mm/h, x±s) | 20.60±13.95 | 28.75±19.64 | 0.402 |
| Pre- Urea(mmol/L, x±s) | 5.26±1.61 | 5.73±2.10 | 0.660 |
| Post- Urea(mmol/L, x±s) | 6.17±1.89 | 8.27±2.45 | 0.113 |
| Pre- Cr(μmol/L, x±s) | 63.40±16.07 | 80.12±37.41 | 0.292 |
| Post- Cr(μmol/L, x±s) | 59.88±14.40 | 79.37±44.89 | 0.284 |
| Pre- eGFR(x±s) | 87.68±12.18 | 74.12±22.06 | 0.182 |
| Post- eGFR(x±s) | 90.74±10.41 | 76.35±24.77 | 0.177 |
| Pre- CysC(mg/L, x±s) | 1.16±0.40 | 1.24±0.0.30 | 0.721 |
| Post- CysC(mg/L, x±s) | 0.97±0.26 | 1.11±0.36 | 0.477 |
| Pre- GGT(IU/L, x±s) | 23.80±8.72 | 27.62±10.74 | 0.498 |
| Post- GGT(IU/L, x±s) | 19.40±4.50 | 34.00±35.38 | 0.285 |
| Pre- ALT(IU/L, x±s) | 16.60±6.22 | 18.37±8.10 | 0.666 |
| Post- ALT(IU/L, x±s) | 14.40±3.50 | 18.37±5.95 | 0.350 |
| Pre- AST(IU/L, x±s) | 17.00±6.59 | 18.37±5.95 | 0.714 |
| Post- AST(IU/L, x±s) | 15.80±4.81 | 18.87±7.56 | 0.390 |
| Pre- RPP(x±s) | 13173±678 | 14031±856 | 0.073 |
| Post- RPP(x±s) | 11456±2815 | 12375±1630 | 0.532 |
| Pre- DP(mmHg, x±s) | 75.60±9.236 | 78.88±7.39 | 0.524 |
| Post- DP(mmHg, x±s) | 67.40±4.72 | 67.75±7.70 | 0.921 |

Data are presented as the mean±standard deviation (SD) and compared by one-way analysis of variance (one-way ANOVA).(*: p＜0.05)


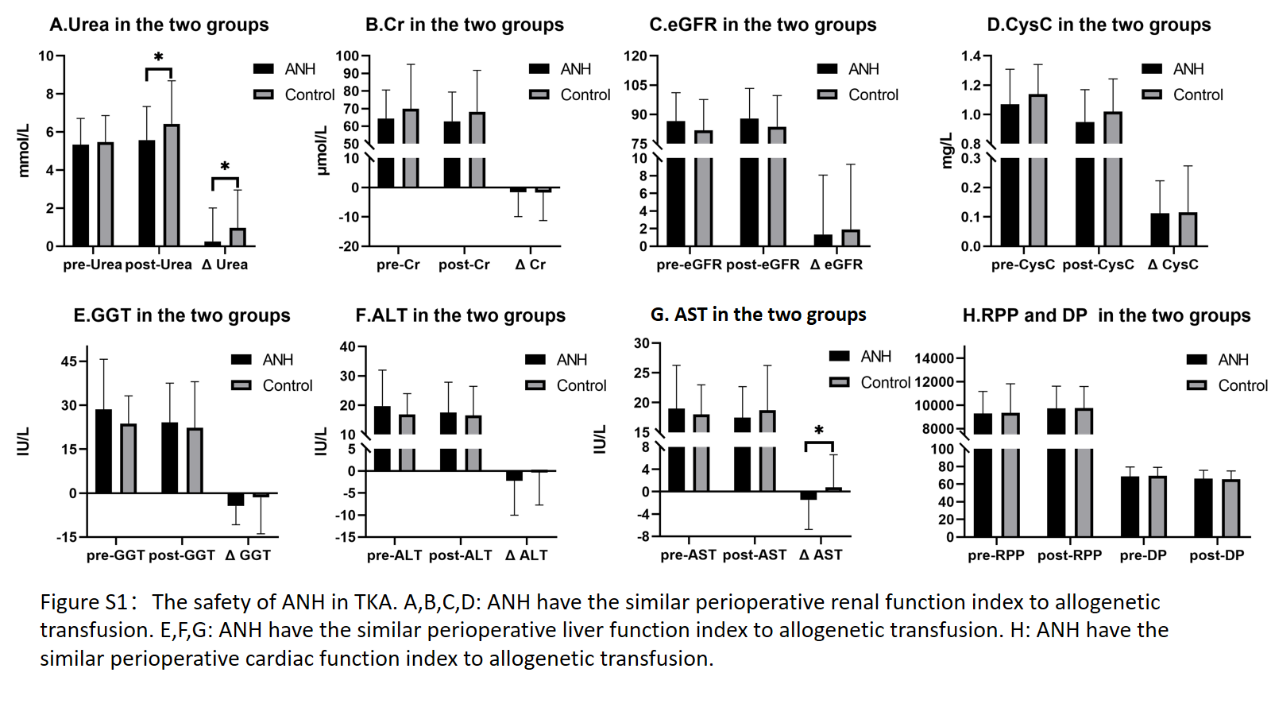


Figure S1： The safety of ANH in TKA. A,B,C,D) ANH have the similar perioperative renal function index to allogenetic transfusion. E,F,G) ANH have the similar perioperative liver function index to allogenetic transfusion. H) ANH have the similar perioperative cardiac function index to allogenetic transfusion. (*: P<0.05)


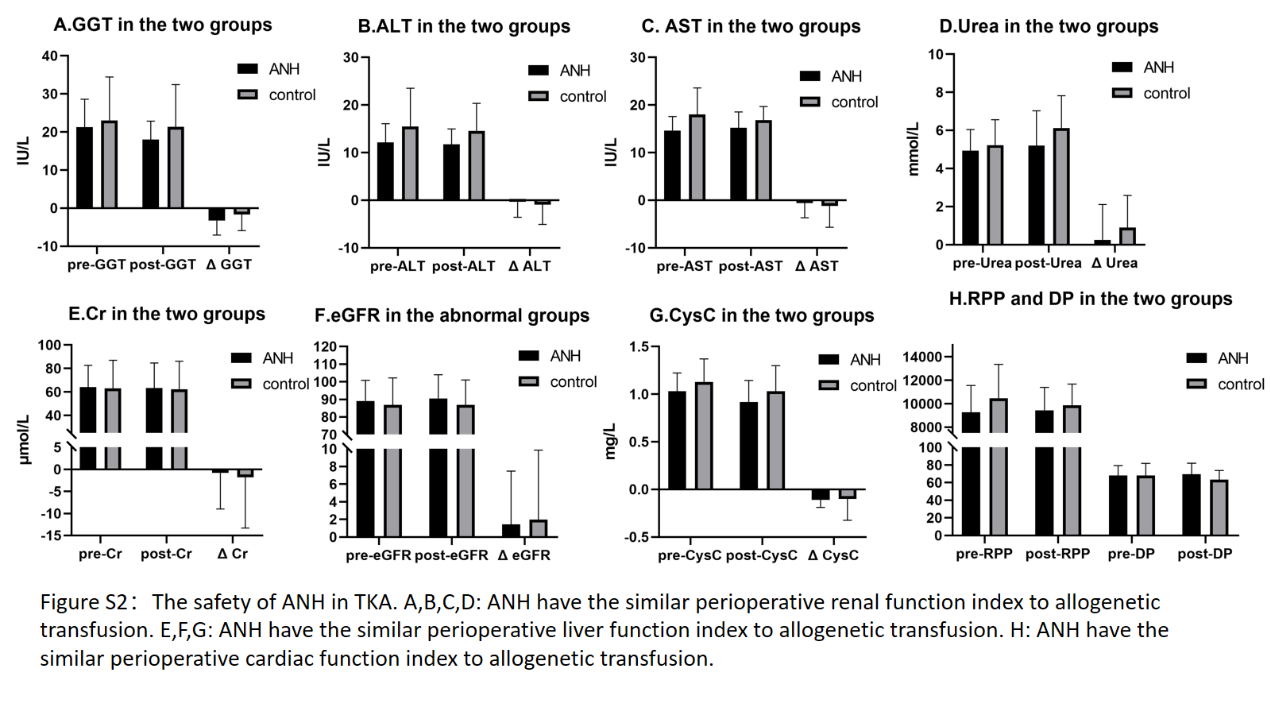


Figure S2： The safety of ANH in TKA. A, B, C, D) ANH have the similar perioperative renal function index to allogenetic transfusion. E, F, G) ANH have the similar perioperative liver function index to allogenetic transfusion. H) ANH have the similar perioperative cardiac function index to allogenetic transfusion. (*: P<0.05)
